# Our New York Letter

**Published:** 1891-08

**Authors:** Wm. L. Russell

**Affiliations:** 151 East 50th Street; New York


### Correspondence.

### OUR NEW YORK LETTER.

NEW YORK, July 15, 1891.

### THE UNIVERSITY.

The Fifty-first Annual Announcement of the Medical Department of the University, which has recently appeared, is particularly interesting from the fact that it heralds a change in the management and curriculum which is of great importance to the advance of medical education in America. This becomes more apparent when it is stated that 6,033 matriculates have been awarded the degree of Doctor of Medicine by the University, since the department was established, 203 of whom graduated at the last commencement. By the new arrangement, the pecuniary interests of the college are placed in the hands of a Board of Trustees, by whom the professors and tutors are paid stated salaries, which are entirely independent of the number of students who matriculate or graduate. A three years' course of study at the college has now been made compulsory instead of optional, as formerly. A still more important change, however, is the subordination of didactic lectures to recitations, and laboratory and clinical work, in the first and second years. The first year's curriculum consists of recitations in anatomy, chemistry, physiology, and pathology, with dissection, and laboratory work in chemistry, materia medica and histology. In the second year lectures are given in experimental chemistry, physics and hygiene, experimental physiology, surgical and regional anatomy, and materia medica ; and recitations are begun in medicine, surgery and obstetrics. Six hours daily for two months of this year are spent by the student in the pathological laboratory, and he attends medical and surgical clinics.

Thus the whole course is gone over in recitations, and a thorough groundwork is given in the principles underlying the art of medicine, before the student listens to a didactic lecture on the more practical subjects of the course.

In the third year, however, lectures are given in medicine, surgery and obstetrics, and in gynæcology, therapeutics and pathology. General and special clinics are also given to the class as a whole, while sections of about twenty-five are given daily instruction at the bedside in Bellevue Hospital, where opportunities are offered for observations of the progress of diseases, and the effects of remedies.

Another good feature of the course consists of an arrangement to insure the attendance of each student upon a number of obstetric cases before his graduation. This does away with the former custom of authorizing men to attend women in confinement before they had made a vaginal examination of a parturient woman, or were in any degree practically familiar with the most ordinary duties devolving upon them as accoucheurs.

The University now offers, I believe, the best course of study of any college in New York, and the fine equipment of all the laboratories, and the abundance of clinical material here, would lead one to believe that no better is at present offered in this country.

### A MEDICO-LEGAL POINT.

A murder trial which has just been completed in this city is of some interest to medical men, from the fact that the expert medical evidence was largely instrumental in deciding the verdict. The facts of the case were somewhat as follows : During the night of April 23d, of this year, an abandoned woman known as "Shakespeare" was found dead and mutilated in a room in a vile resort called the East River Hotel. It was apparent that she had been murdered, and the mutilation of the body suggested the possibility of the murderer being "Jack the Ripper," of Whitechapel notoriety. The body of the murdered woman was found on the bed, with a blood-stained shawl and petticoat wrapped around the head. The lower part of the abdomen had been opened and a portion of the ileum and one ovary cut out. A blood stained knife was found near the body, and the ticking of the bed was soaked in blood. It was also discovered that there were blood stains on the floor in the hall, and bloody finger-marks were seen by the side of the door of the room opposite that in which the body was found, and inside the door on the wall. There was, also, a spot of blood as large as a dollar on the bed in this room.

It was learned that " Shakespeare " had gone to the room in which she was murdered accompanied by a man, who disappeared during the night, and has not been heard of since. The opposite room had been occupied by a man, an Algerian by birth, known as " Frenchy." This man, " Frenchy," was arrested near the hotel the night after the murder. His socks were stained with blood, as were, also, the front flap and sleeves of his shirt. His nails were very long, and four days after his arrest the matter beneath them was removed and preserved for examination.

It was stated at the trial that the murdered woman had eaten corned-beef and cabbage during the day of April 23. Microscopical examination of the various blood stains and of the matter taken from beneath the prisoner's nails was made by Dr. Henry F. Formad of the University of Pennsylvania, Dr. Cyrus Edson of the Health Department of this city, and Dr. Austin Flint. On the strength of their discoveries it was declared that the blood on " Frenchy's" clothing was mixed with the contents of the small intestine. All the evidence against the prisoner being circumstantial, this became a very important point, especially as all the other witnesses except the experts were degraded characters, whose words were untrustworthy.

It was stated by the experts that the blood stains examined were of mammalian blood. In the specimens from the back of the prisoner's shirt, from one sleeve, from the floor of the hall, and from the ticking of the woman's bed, her stockings and petticoat, only blood was found. In the matters from under the prisoners nails, the front flap and right sleeve of his shirt, and from the wall-paper of the hall, the blood of the door and casing, the socks of the prisoner, and the bedding of the room which he occupied, blood was found mixed with biliary coloring matter unchanged; fat globules and crystals, tyrosine, cholesterin, triple phosphates columnar epithelium, eggs of round worms, starch granules, partially digested muscular tissue, and vegetable matters.

In an article in the last number of the *New York Medical Journal*, Dr. Flint states that, in the specimens examined by him, the most prominent features were in addition to the blood, the sheaves of crystals of tyrosine, columnar epithelium stained with bile, the partially digested muscular tissue, and a very few muscular fibres nearly perfect in structure, with the hard residue of spiral and other vegetable cells. The presence of tyrosine, of bilirubin, and the very slightly altered muscular fibres led him to the belief that the matters were from the small intestine.

He testified to this effect, and that the specimens presented practically the same appearance. He did not believe that the contents of the large intestine (fæces) would show tyrosine or bilirubin. Dr. Formad gave a similar opinion, and stated emphatically that he would be willing to stake his life on its soundness.

On these grounds and that the large intestine of the woman had not been injured, the jury were convinced that the prisoner was guilty, and brought in a verdict of murder in the second degree. This is one of the most remarkable trials that has ever been held here, and the medical testimony has provoked much discussion.

### INDIGESTION A CAUSE OF INSOMNIA.

This is the subject of an editorial in a recent number of the Boston Medical and Surgical Journal. It is stated that in practice among children, the physician invariably seeks for some disturbance of digestion when insomnia is complained of. In certain persons, too, it is well known that to eat even a moderate meal late in the evening means a sleepless night. The flatulence produced by indigestion distends the stomach which presses on the thoracic organs and causes disagreeable sensations and palpitations. Toxic products are also formed which irritate the nerve centres and render the cerebrum hyperæmic.

Persons with healthy stomachs and normal arterial tone on the other hand, find that sleep is not in the least disturbed by the process of digestion. In fact many of them sleep readily and comfortably after a full meal. It is denied that the digestive functions are practically suspended during sleep, as is claimed by some authorities. The functions of the stomach and intestines are continued during sleep though with lessened activity ; the secretions are not suspended, and the unstriped muscular fibres continue to act, though sluggishly. In fact, all the essential functions, such as circulation, respiration, digestion, etc., continue to be exercised during sleep but not so actively.

Most cases of digestive insomnia are due to difficulties attending the secondary or intestinal digestion. The patient goes to bed at the usual hour, but is unable to sleep for hours. The wakeful period corresponds with the time of pancreatico-intestinal digestion of his last meal, and when the process is completed he goes to sleep. That this is true can be proved by having him eat only a very light supper, or eat nothing after four o'clock, when he will go to sleep at the usual time.

The treatment of these cases requires careful attention to the quality and quantity of the food. Errors as to quantity are most frequent and must be avoided. Proper cooking is an important matter, and a judicious selection of meat that is juicy and tender. Men of sedentary habits, "brain-workers," constitute the largest proportion of sleepless patients, and it is very important to them especially that food should not be difficult of digestion. The laborer working his muscles in the open air needs foods that digest slowly, and his sleep is not disturbed in the slightest degree by boiled beef and cabbage, pork and beans or mince-pie. Not so with the man of sedentary habits, however, who should avoid or eat but sparingly of food known to be difficult of digestion.

Physical exercise, a cold bath every morning, change of scene and diversions, and the cultivation of a contented, cheerful frame of mind are important points in the treatment of many cases.

Among medicinal agents, alkalies before meals, and acids and pepsin after, have been found valuable. Small doses of strychnine are useful. Professor Germain Sée advises sufferers from acid dyspepsia to take a drachm of bicarbonate of soda in hot water at bedtime. Constipation is usually present, and requires such laxatives as rhubarb, Glauber salts, cascara, enemata of hot water, etc. The last mentioned often proves a sovereign remedy for insomnia. When there is a torpid liver, euonymin, podophyllin, or blue pill is indicated.

WM. L. RUSSELL.

151 *East* 50*th Street.*

